# A Review on Image Processing and Fractal Analysis in Oral Potentially Malignant Disorders

**DOI:** 10.1111/odi.70271

**Published:** 2026-02-26

**Authors:** André Goulart Poletto, Flaviana Silva de Souza, Lilian Bezerra, Thiago Pires Claudio, Jesse Parente Tocantins, Helena Miguel Cotter, Riéli Elis Schulz, Karin Berria Tomazelli, Elena Riet Correa Rivero, Fabio Luiz Coracin, Gustavo Davi Rabelo

**Affiliations:** ^1^ Department of Dentistry Federal University of Santa Catarina Florianópolis Santa Catarina Brazil; ^2^ Department of Pathology Federal University of Santa Catarina Florianópolis Santa Catarina Brazil; ^3^ Department of Dentistry and Pathology Barretos Cancer Hospital Barretos São Paulo Brazil

**Keywords:** artificial intelligence, computer vision systems, intraoral photography, leukoplakia, oral cancer

## Abstract

**Aim:**

The clinical evaluation of patients with oral potentially malignant disorders is primarily based on physical examination and observable clinical features. Clinical photographs play a key role in patient monitoring and help identify signs that may indicate malignant transformation. These images can be accessed by artificial intelligence techniques and fractal analysis, and image processing and segmentation are crucial for these tasks. This review discusses image processing techniques applied to clinical photographs of oral potentially malignant disorders, with emphasis on fractal analysis.

**Methods:**

Studies investigating fractal dimension and lacunarity as measures of lesion complexity and homogeneity were reviewed, along with research on segmentation and binarization.

**Results:**

A search of scientific databases identified 10 studies reporting the access of texture parameters on clinical images to measure fractal dimension and lacunarity.

**Conclusions:**

The reviewed studies showed that fractal dimension and texture parameters were useful for evaluating lesions and distinguishing normal and pathological tissues; however, there is a lack of methodological standardization. Although fractal dimension was assessed in all articles, lacunarity was examined in just one. Binarization and segmentation impact fractal dimension, lacunarity, and other texture measurements, which can be significantly enhanced by appropriate image processing. In contrast, poorly executed preprocessing may lead to misinterpretation of results.

## 1 Introduction

Evaluation of oral potentially malignant disorders (OPMD) in clinical practice usually focuses on identifying the principal features related to the physical aspects of the lesion, particularly in oral leukoplakia (OL), where these features are also used for classification (De‐Abreu et al. 2023). The last consensus organized by the World Health Organization Collaborating Centre for Oral Cancer summarized the clinical presentation of the most important OPMD lesions, and regarding OL, there are two major types: homogeneous and non‐homogeneous. The non‐homogeneous OL are divided into three subtypes: nodular, verrucous, and erythroleukoplakia. Other lesions in the OPMD group include oral erythroplakia, proliferative verrucous leukoplakia, oral lichen planus (OLP), oral submucous fibrosis, oral lichenoid lesion, and oral graft‐versus‐host disease (Warnakulasuriya et al. 2021). Clinical presentation of these lesions is used in their evaluation and monitoring (Ilhan et al. 2021; Vinayahalingam et al. 2024), and photography is considered one of the most powerful tools in this task. Through photographs and their corresponding digital images, it is possible to assess changes in the appearance of the lesion, including alterations in texture, color, physical presentation, size, and shape, among many other characteristics (Witt et al. 2024). Also necessary, photographic documentation of lesions is valuable for screening, referring patients to specialists, and facilitating telehealth and teleconsultation (Carrard et al. 2018; Flores et al. 2022).

Intraoral and extraoral photographs can be used beyond clinical evaluation and monitoring, and computer vision algorithms can be applied to perform more in‐depth analysis of image data, mostly on feature extraction. Fractal Analysis (FA) can be interpreted as an alternative to assess the homogeneity and complexity of a structure, and two indices can be extracted from an image: fractal dimension (FD) and lacunarity (Lac) (Irie et al. 2018; Santos et al. 2023; Tomazelli et al. 2025). By definition, FD can be characterized as the numerical representation of FA, assessing the structural organization of a given structure through measurements of its spatial occupation and morphological complexity (Sánchez and Uzcátegui 2011). On the other hand, Lac is the counterpart of FD, which numerically represents the distribution of empty spaces and *invariation* to the translational rotation, meaning that it is the measure that evaluates the considered “gaps” in an image regarding their localization and distribution (Dong 2000; Tomazelli et al. 2025).

Preprocessing techniques are reported to be essential in image analysis and for preparing data for machine learning (ML) and AI methods, which can precede FA and other texture feature evaluation. A recent literature review on the topic showed that AI processing/preprocessing methods (focusing on data structure, preprocessing, and data organization), with particular relevance to cancer diagnosis, have highlighted the need for careful consideration and caution when interpreting the outcomes (Araújo et al. 2025). Preprocessing can be divided into steps that enhance image quality and support the segmentation process. These steps or methods are focused on their tasks according to the outcome needed, such as image resizing or fragmentation, segmentation techniques, normalization/standardization methods, data augmentation, and noise reduction (De‐Abreu et al. 2023; de Souza et al. 2023; Araújo et al. 2025), but little is known about feature extraction regarding FA.

Despite numerous studies on OPMD, concern persists regarding the use of clinical images for diagnostic purposes. A recent literature review with meta‐analysis on the diagnostic performance of AI in detecting OPMD and oral cancer highlighted a critical limitation: many studies did not follow standardized guidelines for preparing image data before training AI models. The authors noted that specific strategies, such as ensuring the lesion is centered in the image to reduce focal‐length distortions, could significantly improve diagnostic outcomes (Sahoo et al. 2024). However, this information might introduce a methodological concern, as consistently placing the lesion at the center could lead the convolutional neural networks (CNN) to “learn” this positional pattern, thereby introducing bias and compromising detection performance. Regarding the application of FA in OPMD images, only five studies to date have used this method, suggesting that the topic remains poorly investigated. Therefore, this study aimed to provide a review of the available literature on image processing techniques applied to clinical photographs of OPMD, with a particular focus on identifying methodological gaps and limitations, especially in binarization, segmentation, and FA. In addition, to go further on the review of tools and processing steps that could enhance the evaluation of these lesions in clinical imaging.

## 2 Literature Review

An electronic search was conducted on June 4, 2025, using two databases—PubMed and IEEE Xplore—employing the following search terms: “Oral potentially malignant” OR “Leukoplakia” AND “Fractal” OR “Lacunarity”, which yielded 19 studies, of which only 5 focused (Spyridonos et al. 2015; Jurczyszyn et al. 2018; Latini et al. 2018; Iqbal et al. 2020; Jurczyszyn and Kozakiewicz 2020) on the analysis of clinical images and were included in the review. A complementary search was conducted on October 9, using the same strategy in the Google Scholar database, which returned 5 studies (excluding PhD and graduation thesis) (Pandey et al. 2015; Nanditha et al. 2020; Nagarajan and Jayachandran 2020; Jurczyszyn et al. 2021; Varsha et al. 2023). In addition, a manual selection of studies addressing binarization and segmentation techniques was performed, as these image processing steps are critical for accurate FA. Considering their importance, the discussion was expanded to emphasize the relevance of preprocessing methods not only for FA but also for AI applications. Due to limited studies, a systematic review was not conducted.

### 2.1 Binarization

**FIGURE 1:**
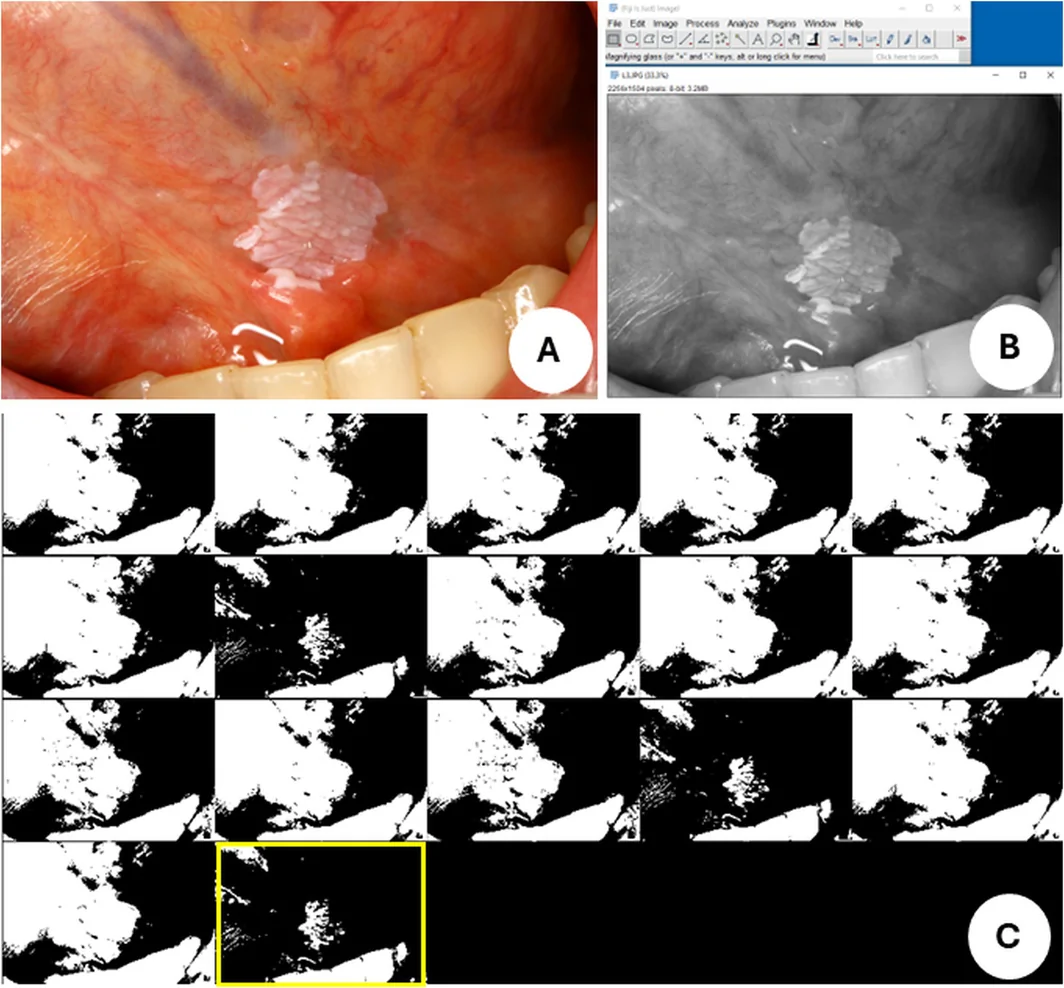
Oral leukoplakia binarization process. (A) Clinical digital image of oral leukoplakia. (B) Same image opened in ImageJ/Fiji software and converted to 8‐bit grayscale. (C) Application of auto‐thresholding methods to the 8‐bit image. In a qualitative assessment of the binarized outputs, only two thresholding methods—MaxEntropy (seventh position) and Yen (seventeenth position)—produced masks that accurately delineated the leukoplakia in the center of the image. The Yen method (highlighted with a yellow square) provided the most suitable binary mask for automated segmentation of the lesion.

Among the several methods to process a digital image in computer vision, one of the most widely used is binarization, which can be defined as the transformation of a digital image into a bi‐level form, where the foreground is represented by black pixels and the background by white pixels, most commonly using a histogram‐based method (Stathis et al. 2008). Three recent important systematic reviews approaching the subject of OPMD and the use of AI models have focused on diagnostic accuracy, sensitivity, sensibility, and in the performance of AI architectures across diverse imaging modalities for the lesion detection. However, only one of these reviews questioned the preprocessing, and none reported the importance of binarization for the final outcomes (Gomes et al. 2023; Sahoo et al. 2024; Li et al. 2024). For instance, the software ImageJ/Fiji offers 17 options for automated image binarization, which can be incorporated into a computational code or an AI model for segmentation or classification. All 17 methods can be applied simultaneously, allowing the operator to choose the best binarization method to include in the preprocessing. Figure 1 shows an image of an OL that the 17 methods of binarization were tested on—some revealing good binarization results, while others did not. According to the manual, the auto‐threshold plugin binarizes 8 and 16‐bit images using various global (histogram‐derived) thresholding methods. The segmented phase is always shown as white (255) (https://imagej.net/plugins/auto‐threshold).

Regarding ML methods in AI, not all of them require binarization when applied to digital images for segmentation, recognition, or classification. Deep learning models, specifically CNN, do not necessarily require binary images and can work with images in color (RGB system) or grayscale, although creating masks is quite common at one point within the model tasks (Shin et al. 2016; Simon and Aliferis 2024; Zatt et al. 2024; Gao et al. 2025). Masks are especially useful in segmentation, localization, or attention‐related tasks (Shin et al. 2016; Simon and Aliferis 2024), and when considering OPMD clinical images, a mask can be applied to filter out unwanted regions or focus on relevant parts of the image, which would be an important step before going on FD and Lac assessment, pursuing to evaluate a more number of images or regions of interest in the same lesion (in case of choosing square regions of interest, which is the most used method in the literature). Considering this approach, Lee et al. (2024) used Otsu's thresholding to convert the Class Activation Mapping, used in the mosaic‐based soft labeling, into a binary map that distinguishes representative from nonrepresentative regions of tongue lesions, including OPMD (Lee et al. 2024). Interestingly, we emphasize that, in our opinion, whether AI models explicitly use binarization or not, the binary mask is essential for obtaining quantitative metrics and measurements, such as FD and Lac. Therefore, when measuring lesions or extracting other characteristics (such as texture, color, and so on), binarization becomes indispensable.

A recent study reported that ML‐based systems have been applied to classify images and validate various analytical methods for the clinical evaluation of OPMD and according to the authors, during the preprocessing and processing phases, key tasks include delineation and annotation (i.e., labeling and delimiting the region of interest in clinical images), semantic segmentation to define lesion boundaries, fragmentation of images into sub‐images, classification, and data augmentation (de Souza et al. 2023). Notably, across all phases and tasks involved in developing AI models, including CNN and other ML architectures, the use of binarization can significantly influence results and final outcomes. Therefore, it is crucial that an Oral Medicine specialist thoroughly verify and evaluate the binarization and other preprocessing steps to ensure they accurately represent the lesion intended for analysis by the AI model. De‐Abreu et al. (2023) highlight the importance of image pre‐processing, encompassing clinical photography, interactive segmentation, and automated binarization, for the analysis of OL using AI. The study emphasizes that these steps are essential to ensure data quality and that image labeling (as homogeneous or non‐homogeneous) still relies on expert consensus.

More specifically, in the context of FA, binarization is a key step when applying methods to calculate FD and Lac, whether applied solely to binarization or, when required, to both binarization and skeletonization. Cotter and Rabelo (2025) discussed these processes and the need for binarization when the goal is to calculate FD based on skeletonization, which is the process of creating a linear skeleton as the graphical representation of a structure in a binary image. In the review of Latini et al. (2018), they report the use of fractal geometry in the analysis of the microvessel architecture of the vascular network in a photographic image of the oral mucosa, so in their study skeletonization was crucial to be representative of the vessel network structure. In the case of true OPMD lesions, one to assume that using skeletonization in OPMD images should be based on the fact that the skeletonized structure would be representative of the lesion's surface and texture, including that the skeletonized linear representation would be linked to the surface variation of the lesion.

In summary, binarization, whether preceded by segmentation or not, refers to the conversion of a grayscale or color image into a binary representation, typically serving as a preprocessing step for subsequent analyses. When applied beforehand, segmentation divides the image into meaningful regions, isolating the region of interest (ROI) from the background or from other areas irrelevant to the analysis.

### 2.2 Segmentation and Related Tasks

In the context of FA, segmentation plays a crucial role in accurately assessing FD and Lac. The ability to separate the lesion from the background in a digital image can significantly influence these measurements. For instance, in grayscale‐based method that do not involve binarization, such as the one using the FracLac plugin (“FracLac for ImageJ”) for the software ImageJ/Fiji (Schneider et al. 2012), the analysis often includes the entire image, not just the lesion, unless segmentation is specifically applied (when it is possible, for example selection a square shaped region of interest (ROI) within the lesion). In other approaches, it is essential to ensure that the segmentation file (e.g., a .roi file generated from the ROI containing lesion contour information) is properly incorporated into the algorithm or macro used to run the analysis. Manual segmentation by an expert operator (interactive segmentation) remains the most accurate method, as it relies on precise delineation of the lesion. However, this process is time‐consuming and not scalable for large datasets. In this context, AI‐based segmentation methods could offer a valuable solution by enabling efficient, reproducible segmentation, thereby facilitating FA analysis.

A very recent article reviewed the literature regarding the use of AI models to evaluate clinical photographs of oral cancer and OPMD reveals the importance of segmentation and describes the ground truth annotations, that is, the task to encompass all annotations made by human experts, will be used to train models in supervised learning and to gauge model performance (Mirfendereski et al. 2025). These tasks to delineate the lesion boundaries are marked to create an orientation map that accurately represents the size, shape, and location within the image. The authors noted that only a few studies reported the use of calibration methods and criteria to address subjectivity in annotations and segmentation (Mirfendereski et al. 2025). Why is this so important to FA? Because FD and Lac assessment would be calculated only in the lesional area of the image, segmentation is crucial in this context. Using AI segmentation models alongside FA would be of great interest, especially given the time‐saving benefits. In this sense, segmentation (interactive or not) would rely on previously classified and/or segmented images through AI‐based systems, which could help perform these tasks on a larger number of images. At this point, classification seems to still be an issue in OPMD studies. In a previous study with 55 images of OL, the five experts in the field of oral medicine did not agree on all images when classifying them as homogeneous or non‐homogeneous (De‐Abreu et al. 2023). Another study by Araújo et al. (2023) with images from 37 patients diagnosed with OPMD revealed that the professional's subjectivity of the involvement in the clinical assessment may have contributed to the discordance in classification and annotation. They reported that subjectivity may be related to variations in human visual observation, variable criteria that have been adjusted over the years, different educational backgrounds, and personal experience. The initial steps—mainly concerning segmentation—are probably the ones that could have the most impact on the sensitivity and specificity levels because with inaccurate lesion boundaries, FA assessment could be compromised, or at least, be more inaccurate.

### 2.3 Fractal Dimension and Lacunarity

**FIGURE 2:**
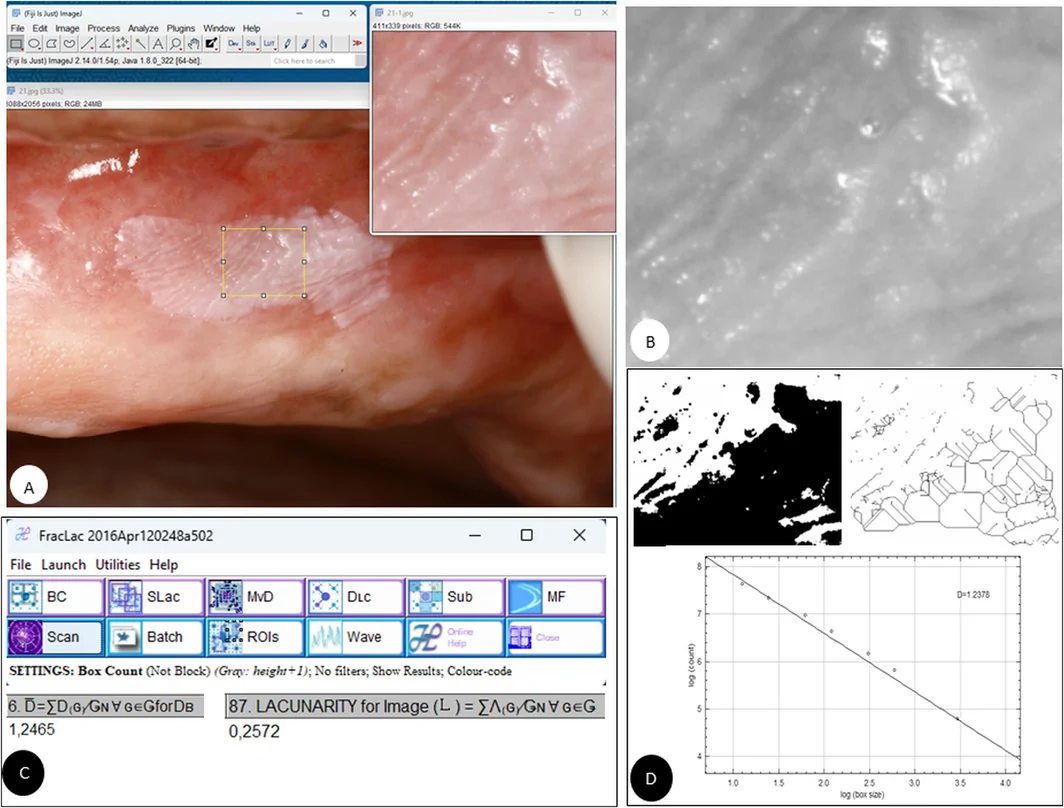
Clinical photograph of oral leukoplakia used for fractal dimension (FD) and lacunarity (Lac) analysis by two different methods. (A) Clinical digital image of oral leukoplakia showing the selected square region of interest (ROI) in the center of the lesion (yellow square) and the magnified ROI in the upper right corner. (B) ROI converted to 8‐bit grayscale. (C) FracLac plugin interface used for the gray‐scale–based analysis of FD and Lac (Karperien 2013). The box‐counting method was applied using the “Gray1: Differential” option (for grayscale images without binarization). The resulting summary file provided FD values (column 6, D) and Lac values (column 87, LACUNARITY for Image (L)). (D) FD assessment using the conventional method: Binary transformation of the ROI (upper left; “Default” binarization), followed by skeletonization (upper right, showing the linear representation of the ROI structure) and FD calculation using the “Fractal Box Count” tool.

Fractal analysis is an objective approach to images‐containing‐objects evaluation, and it is especially effective when the objects exhibit irregular shapes arranged in self‐similar structures across multiple scales (Lopes and Betrouni 2009), such as the OPMD lesions in photographs, revealing their color and texture variations. The 10 articles identified that applied FA to the clinical evaluation of OPMD adopted different methodological approaches. These included the use of FD and related parameters to assess lesion characteristics—mainly OL—and to distinguish normal from pathological conditions, such as OL and OLP, comparisons between lesional and non‐lesional mucosa, pre‐ and post‐treatment evaluation, and differentiation between two white lesions. All 10 studies investigated FD, whereas only one specifically analyzed Lac. The assessment of FD and Lac by two distinct methods is illustrated in Figure 2: one based on image binarization and skeletonization (for FD calculation), and the other on gray‐scale pixel distribution analysis (for both FD and Lac calculation). Table 1 summarizes the objectives, methodological variations, and main findings of the ten included studies.

**TABLE 1:** Aims, methodological aspects and main findings of the 10 studies included in the review.

Author (year)	Objectives	Method			Main findings
		Binarization	Segmentation	FD	
Pandey et al. (2015)	To compare pretreated and post‐treated OL.	White and Rudolph method.	Square ROIs 86 × 124 pixels.	Box counting method using ImageJ (NIH, USA).	The difference between pre‐ and post‐treatment was statistically significant. Although the treatment was not specified in the original article.
Spyridonos et al. (2015)	To analyze the degree of border irregularity in patients with actinic cheilitis compared to a control group.	The images were binarized only on FBC2 method—the information about binarization is not clearly specified.	Clustering algorithmic Fuzzy C‐means (automated segmentation method).	Three different methods of FD assessment were applied using the software MATLAB (2010, The Mathworks, USA) and compared: two utilizing the box‐counting method (FBC1 and FBC2) and one using the Sevcik approximation.	The box‐counting grid method (FBC2) was considered the most appropriate morphological descriptor for measuring irregularities in patients with actinic cheilitis. The other two methods were not as effective in irregularity analysis.
Latini et al. (2018)	To review clinical biomarkers for cancer recognition and prevention.	The images underwent processing to threshold the vessel network and remove background interference. Subsequently, the resulting networks were skeletonized.	Square ROIs of 251 × 251 pixels.	Box‐counting method using Image Pro‐Plus (Media Cybernetics Inc., Silver Spring, USA).	The authors mention a pilot study in which the FD of microvessel distributions on the oral mucosa was significantly higher in patients with hereditary nonpolyposis.
Jurczyszyn et al. (2018)	To distinguish OL from OLP through FD in pictures obtained with white light and through PDD.	Application of filters and histogram equalization; images converted to grayscale and binarized.	Square ROIs of 300 × 300 pixels at the center of the lesion.	Box‐counting method using the Fractalyse software v.2.4 (University of Franche‐Comté, Besançon, France).	FD did not significantly differ between OL and OLP using the conventional white‐light. When comparing the lesion site, there was a significant difference in OLP between cheek vs. alveolar ridge. In OL significant differences were found between cheek vs. tongue, alveolar ridge vs. palate and alveolar ridge vs. tongue.
Iqbal et al. (2020)	To determine the efficacy of FD in detecting malignancy potential of oral leukoplakia.	White and Rudolph method.	Square ROIs of 86 × 124 selected in the areas with higher toluidine retention.	Box‐counting method using ImageJ v.1.47 (NIH, USA).	FD was significantly higher in OL with confirmed epithelial dysplasia, both before and after toluidine blue staining. There was also a positive correlation between FD, age, and the duration of tobacco use.
Jurczyszyn and Kozakiewicz (2020)	To apply texture analysis and FD to estimate the effectiveness of OL treatment using an Er:YAG laser.	Images were converted to grayscale and after converted to binary (threshold 137/255).	Square ROI of 150 × 150 pixels at the center of the lesion.	Box‐counting method using Fractalyse software v.2.4 (University of Franche‐Comté, Besançon, France).	No significant difference was found in FD among the reference, intermediate period, and post‐operation groups.
Nagarajan and Jayachandran (2020)	To compare the clinical and therapeutic effects of proton pump inhibitor therapy and vitamin‐A therapy in OL, through FD.	White and Rudolph method.	The information about segmentation was not specified in the original article.	Box‐counting method d using ImageJ v.1.46r (NIH, USA).	No significant difference was found in FD among pre‐ and post‐treatment.
Nanditha et al. (2020)	To analyze fractal features of oral images for lesion identification.	The information about binarization was not clearly specified.	ROI's of 32 × 32 pixels using Particle Swarm Optimization and Seeded Region Growing (automated segmentation method).	Box‐counting method. The software used was not specified in the original article.	FD and lacunarity were significantly higher in malignant lesions.
Jurczyszyn et al. (2021)	To evaluate the effectiveness of photodynamic therapy vs. topical steroid therapy in OLP using novel carriers for the photosensitizer and steroids through FD.	Binarization was not performed, pictures were converted to grayscale, the method analyzes intensity differences between pixels within boxes, working on gray‐level variations rather than binary images.	ROIs 200 × 200 pixels (gingival lesions) or 300 × 300 pixels (cheeks and tongue).	Intensity difference box‐counting method, using the software ImageJ v.2.5 (NIH, USA) and the plugin FracLac.	Under white light, significant changes in FD were observed in pre‐treatment vs. healthy mucous membrane; no significant difference was detected between pre‐treatment and post‐treatment groups.
Varsha et al. (2023)	To compare pre‐ and post‐treatment of OLP, with VAS—burning sensation, FA, and TS—clinical score—size.	White and Rudolph method.	The information about segmentation was not specified in the original article.	Box‐counting method using ImageJ (NIH, USA).	There was a significant reduction in FD after treatment. Paired analysis showed a correlation between the reduction in FD, clinical improvement and pain reduction.

Abbreviations: FA, fractal analysis; FBC, fractal box counting method; FD, fractal dimension; NIH, National Institute of Health; OL, oral leukoplakia; OLP, oral lichen planus; PDD, photodynamic diagnosis; ROI, region of interest; TS, Thongprasom Score; USA, United States of America; VAS, Visual Analogue Score.

A recent comprehensive review of FA applied to the diagnosis of oral cancer and OPMD (Contaldo et al. 2024) revealed that the 27 eligible studies were published between 1993 and 2022. The samples used to FA comprise histological specimens, clinical pictures, radiological images, salivary samples, and cytobrushes, with the main application of the FD performed to improve the classification of histopathological images. Some of the studies included in our article were also included in their review, and they included one more article on OL clinical pictures—among their 27 articles, six were found to include clinical images for FA assessment. Interestingly, the authors discussed that image processing was consistently more accurate and precise in performing textural analysis in the RGB channel; and that in the following steps, there is a transformation into grayscale and later into binary images for better definition through semi‐automatized and automatized image analyses with neural network support.

## 3 Discussion

The accuracy of texture analysis and feature extraction is highly dependent on image preprocessing and processing steps, such as binarization and segmentation. While more precise methods can improve results, they are often more time‐consuming, presenting a practical challenge for large datasets. Furthermore, the classification of OPMD remains a complex challenge. To improve diagnostic accuracy, a comprehensive approach is necessary that integrates precise image annotations with clinical data and robust texture features such as FD and Lac. Our review supports the value of this approach. Among the 10 studies that employed FA using FD and Lac, eight reported significant differences in group and subset comparisons. Specifically, FD proved effective in distinguishing normal from pathological tissue, evaluating OPMD pre‐ and post‐treatment, differentiating between OPMD types, and characterizing texture differences across various oral sites. Lac is still not fully explored because it was used in only one study.

The accuracy of AI models in identifying and classifying OPMD, including the ability to distinguish benign from malignant lesions, as well as predicting cases with potential for malignant transformation, can be significantly enhanced when binarization and segmentation methods are aligned with the specific research question and when the segmented images are used to extract texture features, such as the FD and Lac. This raises an important consideration: will clinical images of OPMD alone, without any other essential data, be suitable for AI models, considering their inherent limitations and potential biases? In this context, incorporating texture analysis through FD and Lac assessments could provide substantial added value by introducing more objective and quantifiable features into the evaluation process. For instance, Iqbal et al. (2020) revealed that FD was significantly higher in OL with dysplastic changes, and both studies of Jurczyszyn et al. (Jurczyszyn et al. 2018; Jurczyszyn and Kozakiewicz 2020) revealed that normal oral tissues were different from lesions, whereas no significant differences between OL and OLP (a tendency of *p* = 0.068 was found in the comparison of the lesions diagnosed in the cheeks). Additionally, lesions from different intraoral sites were differentiated, revealing that the homogeneity of the distribution of the lesion's components (white or red parts analyzed in white light photographs) significantly differed when comparing the cheek, tongue, alveolar ridge, and palate. We believe that FD has already demonstrated effectiveness in the evaluation of lesions and would be valuable in the analysis of OPMD. Its counterpart, Lac, which provides complementary information on texture and structural variation, has been explored in just one study (Nanditha et al. 2020) included in our review, and was effective in characterizing benign and malign lesions. In their study, both FD and Lac were extracted from clinical images (calculated considering the number of self‐similar (invariant) shapes, scaling factor, and the number of gray levels in the image) and revealed higher values for malignant lesions. Our review findings demonstrate that Lac effectively complements FD in discriminating between oral conditions based on color and shape variations in clinical photographs. In another context, studies involving clinical photographs of lesions, excluding OPMD, FD have already proven to be a valuable parameter for quantifying complexity and irregularity. For instance, in a study evaluating 39,270 dermatological lesions, FD was shown to aid in melanoma diagnosis, with a sensitivity of 72.4% and a specificity of 50.1% in distinguishing benign from malignant lesions. When comparing melanoma and non‐melanoma lesions, the sensitivity was 72.8% and the specificity was 50%. The authors concluded that FD alone was sufficient to generate unsupervised AI models capable of effectively classifying dermatological lesions (Romero‐Morelos et al. 2024).

Beyond the helpfulness of texture analysis in clinical evaluation of OPMD, it should be noted that its use works as one more option among several tools available to oral medicine specialists and other healthcare professionals in the evaluation of lesions. The experts would be the ones continuously enrolled as the central part in studies with OPMD, being the ones to take care of labeling, annotations, and choosing the segmentation methods and ROIs, together with other processing methods, which will be decisive in each study. Araújo et al. (2023) already reported that interpretation and manual annotation of clinical images can vary significantly among oral medicine professionals and that could influence the final outcomes of lesion segmentation. In this context, we believe that not just in the segmentation but also in choosing the right areas and regions of interest for fractal evaluation would be crucial in obtaining accurate results, meaning that the oral medicine expert would be the one to guide in the methodology and in choosing image areas and features that should be extracted. Our review found that while six studies employed manual segmentation—most of which selected a central area of the lesion—only two used automated methods (AI‐assisted). This underscores that segmentation remains an expert‐driven task, as choosing an appropriate region of interest is crucial for subsequent analysis. Regarding FD calculation, four studies adapted a step‐by‐step method designed initially for radiographic analysis (White and Rudolph 1999). We posit that this method is suboptimal for clinical images. The preprocessing steps developed for periapical radiographs, such as brightness adjustments to remove large‐scale variations and the removal of all fine‐scale and medium‐scale structure to retain only significant variations in density, are unnecessary. Therefore, we propose a simplified FD evaluation method, as illustrated in Figure 2. Our protocol involves a single binarization step followed by skeletonization. This process generates a graphical linear structure representing the lesion surface, which is then used for FD measurement, providing a more direct and appropriate analysis for clinical lesion photographs.

In the systematic review of studies with a specific focus on evaluating the performance of AI architectures across diverse OPMD and oral cancer imaging modalities, the authors revealed lower values of diagnostic odds ratios for photographic images compared to studies in histopathology and optical coherence tomography, and a sensitivity of 0.82 (Sahoo et al. 2024). All the points regarding binarization and segmentation account for lower sensitivity in clinical images. At one point, it can be suggested that adding more criteria to the definition, classification, and boundary‐setting of OPMD lesions in photographs may improve levels of sensitivity in future studies. As reported by Li et al. (2024), standardized datasets of oral mucosal lesion images to ensure the accuracy and generalizability are necessary, and so, including the major questions on segmentation would be appropriate to reach better outcomes. A recent article by Rajendran et al. (2023) describes the development of a platform for image collection and annotation that was created and resulted in a multi‐sourced international image dataset of oral lesions, which can be assessed for FA.

A critical limitation of this study is the small number of eligible articles included. Due to the limited availability of studies specifically addressing FA in clinical photographs of OPMD, only 10 articles met the inclusion criteria. As a result, it was not feasible to conduct a systematic review or perform a quantitative synthesis such as a meta‐analysis. This limitation highlights the need for more research in this area to enable more robust evidence synthesis in the future.

In conclusion, FA, particularly the assessment of FD and Lac, is a valuable tool for evaluating clinical photographs of OPMD. Our review confirms that these metrics effectively differentiate between lesion types and distinguish pathological from normal mucosa by quantifying textural complexity and spatial organization. To ensure accuracy, lesion segmentation remains a critical, expert‐dependent task. While AI‐based methods show promise, they currently risk introducing error; therefore, expert‐driven segmentation is recommended, at least during the crucial labeling and annotation phases of model development. Ultimately, integrating robust fractal analysis into AI‐supported workflows offers a powerful path toward more objective, reproducible, and accurate diagnostic evaluation of OPMD.

## Author Contributions

**André Goulart Poletto:** writing – original draft. **Flaviana Silva de Souza:** writing – original draft. **Lilian Bezerra:** writing – original draft. **Thiago Pires Claudio:** writing – original draft. **Jesse Parente Tocantins:** writing – original draft. **Helena Miguel Cotter:** writing – original draft. **Riéli Elis Schulz:** writing – original draft. **Karin Berria Tomazelli:** writing – review and editing. **Elena Riet Correa Rivero:** writing – review and editing. **Fabio Luiz Coracin:** writing – review and editing. **Gustavo Davi Rabelo:** conceptualization, supervision, writing – original draft, writing – review and editing.

## Funding

This research was supported by the National Council for Scientific and Technological Development of Brazil (Conselho Nacional de Desenvolvimento Científico e Tecnológico—CNPq), under Grant Agreement No. 403656/2021‐4. The authors Riéli Elis Schulz, Helena Miguel Cotter and Flaviana Silva de Souza also acknowledge support from the Fundação de Amparo à Pesquisa e Inovação do Estado de Santa Catarina (FAPESC).

## Conflicts of Interest

The authors declare no conflicts of interest.

## Data Availability

The data that support the findings of this study are available on request from the corresponding author. The data are not publicly available due to privacy or ethical restrictions.
